# Aspirin enhances sensitization to the egg-white allergen ovalbumin in rats

**DOI:** 10.1371/journal.pone.0226165

**Published:** 2019-12-05

**Authors:** Takahiro Fukushima, Tomoharu Yokooji, Taiki Hirano, Yuta Kataoka, Takanori Taogoshi, Hiroaki Matsuo

**Affiliations:** 1 Department of Pharmaceutical Services, Graduate School of Biomedical and Health Sciences, Hiroshima University, Hiroshima, Japan; 2 Department of Frontier Science for Pharmacotherapy, Graduate School of Biomedical and Health Sciences, Hiroshima University, Hiroshima, Japan; Mie Daigaku, JAPAN

## Abstract

Enhancement of oral absorption of food allergens by non-steroidal anti-inflammatory drugs (NSAIDs), especially aspirin, is considered an exacerbating factor in the development of food allergies. In this study, we examined the effect of aspirin on oral sensitization to and absorption of the egg-white allergen ovalbumin (OVA) in rats. The absorption of OVA was evaluated by measuring the plasma concentration of OVA after oral administration by gavage. To evaluate oral sensitization to OVA, plasma levels of immunoglobulin (Ig) E and IgG_1_ antibodies (Abs) specific to OVA were determined by enzyme-linked immunosorbent assay after initiation of sensitization. High-dose aspirin (30 mg/kg) increased oral OVA absorption and plasma levels of OVA-specific IgE and IgG_1_ Abs compared with those observed in vehicle-treated rats. In contrast, low-dose aspirin (3 mg/kg) exerted no changes in either absorption or sensitization. Spermine, an absorption enhancer, increased the oral absorption of OVA to nearly the same extent as high-dose aspirin, whereas the plasma levels of OVA-specific IgE and IgG_1_ Abs exhibited no significant differences between spermine- and vehicle-treated rats. Among the NSAIDs, diclofenac and indomethacin increased sensitization to OVA, similar to high-dose aspirin, but meloxicam exerted no effects on Ab levels. In conclusion, we showed that high-dose aspirin enhanced oral sensitization to OVA. Our study suggests that enhanced oral sensitization to OVA cannot be ascribed to increased absorption of OVA from the intestinal tract. Although the mechanisms underlying this enhancement of sensitization are still controversial, our study suggests that modification of cytokine production due to impairment of the intestinal barrier function and inhibition of cyclooxygenase-1 activity by aspirin may be involved.

## Introduction

Food allergy is defined as an adverse immune reaction to certain foods. The prevalence of food allergies has been increasing rapidly and is becoming a healthcare problem worldwide. In Japan, the prevalence of food allergies is estimated to be 5–10% in infants (aged 0–6 years) and 1–2% in school-aged children (6–15 years) based on data from epidemiological surveys [1,2]. Various foods, such as peanuts, tree nuts, hen eggs, cow milk, wheat, shellfish and soy, can cause allergic reactions. Among these foods, hen eggs are the most frequent causative food of food allergies in Japan [1,2]. Allergic reactions to foods are induced by specific immunoglobulin (Ig) E-mediated, non-IgE-mediated (cell-mediated), and both IgE and cell-mediated mechanisms. In particular, IgE-mediated allergic reactions are the most common mechanism of food allergies such as immediate-type and food-dependent, exercise-induced anaphylaxis. The pathogenesis of IgE-mediated food allergies is divided into two phases, sensitization and elicitation. In the sensitization phase, an IgE antibody (Ab) specific for an allergen, which enters the body through the gastrointestinal tract, skin, or mucosa, is produced under T-helper type (Th) 2 cell-dominant conditions. Parts of the IgE Ab bind to IgE receptors on the surface of mast cells and basophils. In the elicitation phase, the same ingested allergen cross-links with IgE Abs bound to receptors, leading to activation of mast cells and basophils. Activated mast cells and basophils release chemical mediators including histamines and leukotrienes by degranulation, resulting in the development of clinical symptoms such as urticaria, dyspnea, diarrhea, and systemic anaphylaxis.

Non-steroidal anti-inflammatory drugs (NSAIDs) inhibit cyclooxygenase (COX) activity, in which prostaglandins are produced from arachidonic acid. Two isoforms of COX have been identified: COX-1 and COX-2. COX-1 is constitutively expressed in normal tissues and is involved in the physiological production of prostaglandins. COX-2 is induced by inflammatory stimulation and modulates the inflammatory and immune responses [3]. Thus, the inhibition of COX-2 by NSAIDs results in anti-pyretic, analgesic, and anti-inflammatory effects, whereas COX-1 inhibition causes gastrointestinal injury. This gastrointestinal injury can increase the intestinal permeation of macromolecules via the paracellular pathway. We previously reported that aspirin increased the absorption of ingested allergens after impairment of the paracellular pathway in rats [4–6]. In addition, aspirin-facilitated absorption of ingested wheat allergen elicited allergic reactions in provocation tests in patients with wheat-dependent, exercise-induced anaphylaxis [7,8]. These findings indicate that aspirin induces and exacerbates IgE-mediated allergic symptoms by facilitation of allergen absorption from the intestinal tract during the elicitation phase. However, the effect of aspirin on the sensitization phase is unknown. We hypothesized that aspirin could also enhance oral sensitization to food allergens by increasing allergen absorption from the intestinal tract. In this study, we examined the effect of aspirin on oral sensitization to an egg-white allergen, ovalbumin (OVA), in rats.

## Materials and methods

### Materials

OVA (grade V), spermine, diclofenac, and meloxicam were purchased from Sigma-Aldrich (St Louis, MO, USA). Aspirin and indomethacin were obtained from Wako Pure Chemicals (Osaka, Japan) and Nacalai Tesque (Kyoto, Japan), respectively. Alum adjuvant (Imject^®^ Alum) was purchased from Thermo Fisher Scientific (Waltham, MA, USA). Horseradish peroxidase (HRP)-conjugated mouse anti-rat IgE (MARE-1) and HRP-conjugated goat anti-rat IgG_1_ were purchased from GeneTex (Irvine, CA, USA) and Bethyl Laboratories (Montgomery, TX, USA), respectively. All chemicals used were of the highest purity available.

### Animals

Male Brown Norway (BN) rats aged 4 weeks were obtained from Japan SLC, Inc. (Shizuoka, Japan). Rats were provided with a standard laboratory diet (MF, Oriental Yeast, Tokyo, Japan) and water *ad libitum*. Rats were maintained in a temperature- and light-controlled environment for more than 1 week prior to experiments. At the end of each experiment, rats were euthanized by decapitation under anesthesia. All experiments involving animals were carried out in accordance with the Guide for Animal Experimentation from the Committee of Research Facilities for Laboratory Animal Sciences of Hiroshima University (approval No. A16-44-3, Hiroshima, Japan).

### Oral administration study

To evaluate the effects of aspirin on the absorption of an ingested allergen, plasma levels of OVA in rats were examined as reported previously [9]. Briefly, after overnight fasting, rats were anesthetized with pentobarbital (30 mg/kg, *i*.*p*.) and cannulated with polyethylene tubing (PE-50) at the femoral artery for blood sampling. Vehicle alone [phosphate-buffered saline (PBS), pH 7.4] or vehicle containing aspirin (3 or 30 mg/kg) was administered orally using a stainless-steel feeding tube. OVA (50 mg/kg) dissolved in PBS (pH 7.4) was administered orally 30 min after treatment. To evaluate the effect of spermine on OVA absorption, a mixture of OVA (50 mg/ml) and spermine (20 mg/ml) was administered orally at a dose of 1 ml/kg. Blood (0.25 ml) was collected at designated time intervals for 3 h via the cannula to determine the plasma concentrations of OVA. Each blood sample was centrifuged, and the plasma sample was stored at −30°C until use. The plasma concentration of OVA was determined using a sandwich enzyme-linked immunosorbent assay (ELISA) kit (Morinaga Institute of Biological Science, Yokohama, Japan) and a Microplate Fluorometer (PerkinElmer, Waltham, MA, USA) at a wavelength of 500 nm for excitation and 520 nm for emission, as described previously [9].

### Oral sensitization study

The oral sensitization study was performed without the use of an adjuvant according to the procedure described by Proust et al. [10] with slight modification. Briefly, rats were orally administered vehicle alone (PBS, pH 7.4) or vehicle containing aspirin (3 or 30 mg/kg), diclofenac (1.5 mg/kg), indomethacin (3 mg/kg), or meloxicam (0.3 mg/kg) using a stainless-steel feeding tube. Then, OVA (50 mg) dissolved in PBS (pH 7.4) was administered orally 30 min after treatment. To evaluate the effect of spermine on oral OVA sensitization, a mixture of OVA (250 mg/ml) and spermine (25 mg/ml) was administered orally at a dose of 200 μl. These immunization procedures were repeated every other day for 8 weeks. Every two weeks after the first immunization, blood (0.3 ml) was collected from the jugular vein to check plasma levels of OVA-specific IgE and IgG_1_ Abs using ELISA.

### Measurement of plasma levels of OVA-specific IgE

To confirm sensitization to OVA, plasma levels of OVA-specific IgE and IgG_1_ Abs were determined using an ELISA according to our previous report with slight modification [8]. Briefly, the wells of ELISA plates (F8 MaxiSorp loose Nunc-Immuno^™^ Modules, Thermo Fisher Scientific Inc., Yokohama, Japan) were coated with 100 μl of OVA (10 μg/ml for IgE Ab and 1 μg/ml for IgG_1_ Ab) dissolved in PBS (pH 7.4) overnight at 4°C. After washing with PBS containing 0.1% Tween 20 (PBS-T) six times, plates were incubated with 1% blocking reagent (Block Ace^®^, DS Pharma Biomedical, Osaka, Japan) for 2 h at room temperature. Then, 100 μl of each sample of rat plasma (diluted 1:10 for IgE Ab and 1:30,000 for IgG_1_ Ab in 1% Block Ace^®^) was added to each well and incubated for 2 h (for IgE) or 1 h (for IgG_1_) at room temperature. After washing with PBS-T, wells were incubated with 100 μl of HRP-conjugated mouse anti-rat IgE Ab (diluted 1:1000 in PBS) for 2 h or HRP-conjugated goat anti-rat IgG_1_ Ab (diluted 1:100,000 in PBS) for 1 h at room temperature. Wells were washed with PBS-T and then incubated with 100 μl of tetramethylbenzidine substrate solution at room temperature. After incubation, the reaction was terminated with 100 μl of 1 M phosphoric acid. Absorbance was measured at 450 nm and compared with that at 630 nm as a reference using a Multiskan GO spectrophotometer (Thermo Fisher Scientific).

### Statistical analyses

Data are displayed as the means ± standard errors of the mean (S.E.). Differences in mean values between groups were assessed using Kruskal-Wallis tests or ANOVA, followed by a post hoc Tukey test, Scheffe's F test, or Student’s t-test. A value of *P* < 0.05 was considered statistically significant.

## Results

### Effects of aspirin and spermine on OVA absorption after oral administration

**Fig 1:**
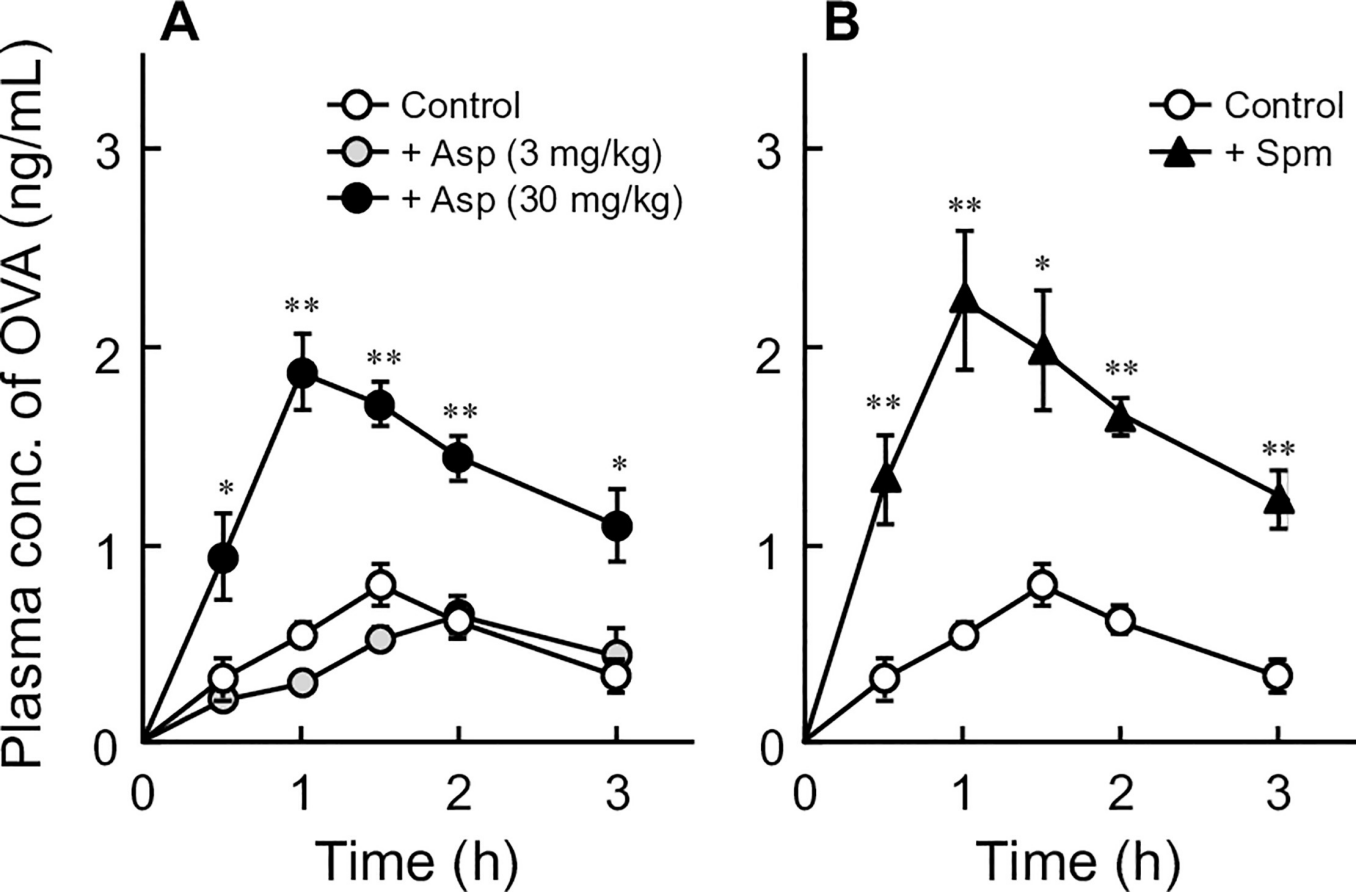
**Effects of aspirin (A) and spermine (B) on plasma concentrations of OVA after oral administration in rats.** Vehicle alone [phosphate-buffered saline (pH 7.4), Control] or vehicle containing aspirin (Asp, 3 or 30 mg/kg) was administered 30 min before oral administration of OVA at a dose of 50 mg/kg. Spermine (Spm, 20 mg/kg) was orally administered with OVA (50 mg/kg) simultaneously. Each value represents the mean ± S.E. of four rats. *P < 0.05 compared with the Control group.

The effects of aspirin and spermine on plasma concentrations of OVA were evaluated after oral administration in rats (Fig 1). The peak plasma concentrations (C_max_) and the area under the concentration–time curves from 0 h to 3 h (AUC_0–3 h_) of OVA are summarized in Table 1. In vehicle-treated (control) rats, oral OVA was absorbed into the blood gradually over time and reached a C_max_ of 0.81 ± 0.09 ng/ml at 1.5 h after gavage. Aspirin increased the OVA absorption in a dose-dependent manner; that is, low-dose aspirin (3 mg/kg) did not affect the plasma concentration of OVA, whereas high-dose aspirin (30 mg/kg) increased the C_max_ and AUC_0-3 h_ by ~2.3-fold and ~3.4-fold, respectively, compared with the values observed in control rats (Fig 1A and Table 1). Spermine increased the C_max_ and the AUC_0-3 h_ by ~2.8-fold and ~4.0-fold, respectively (Fig 1B and Table 1).

**Table 1:** Effects of aspirin and spermine on absorption parameters of OVA after oral administration in rats.

	Control	Aspirin		Spermine
		3 mg/kg	30 mg/kg	
C_max_ (ng/mL)	0.81 ± 0.09	0.61 ± 0.12	1.85 ± 0.19^a^	2.23 ± 0.35^b^
AUC_0–3 h_ (ng h/mL)	1.14 ± 0.13	1.17 ± 0.14	3.84 ± 0.22^b^	4.61 ± 0.32^b^

C_max_, peak plasma concentration; AUC, area under the plasma concentration–time curve. Vehicle alone [phosphate-buffered saline (pH 7.4), Control] or vehicle containing aspirin (3 or 30 mg/kg) was administered 30 min before oral administration of OVA at a dose of 50 mg/kg. Spermine (20 mg/kg) was orally administered with OVA (50 mg/kg) simultaneously. Each value represents the mean ± S.E. of four rats.

^a^*P* < 0.05

^b^*P* < 0.01 compared with the Control group.

### Effects of aspirin and spermine on oral sensitization to OVA

**Fig 2:**
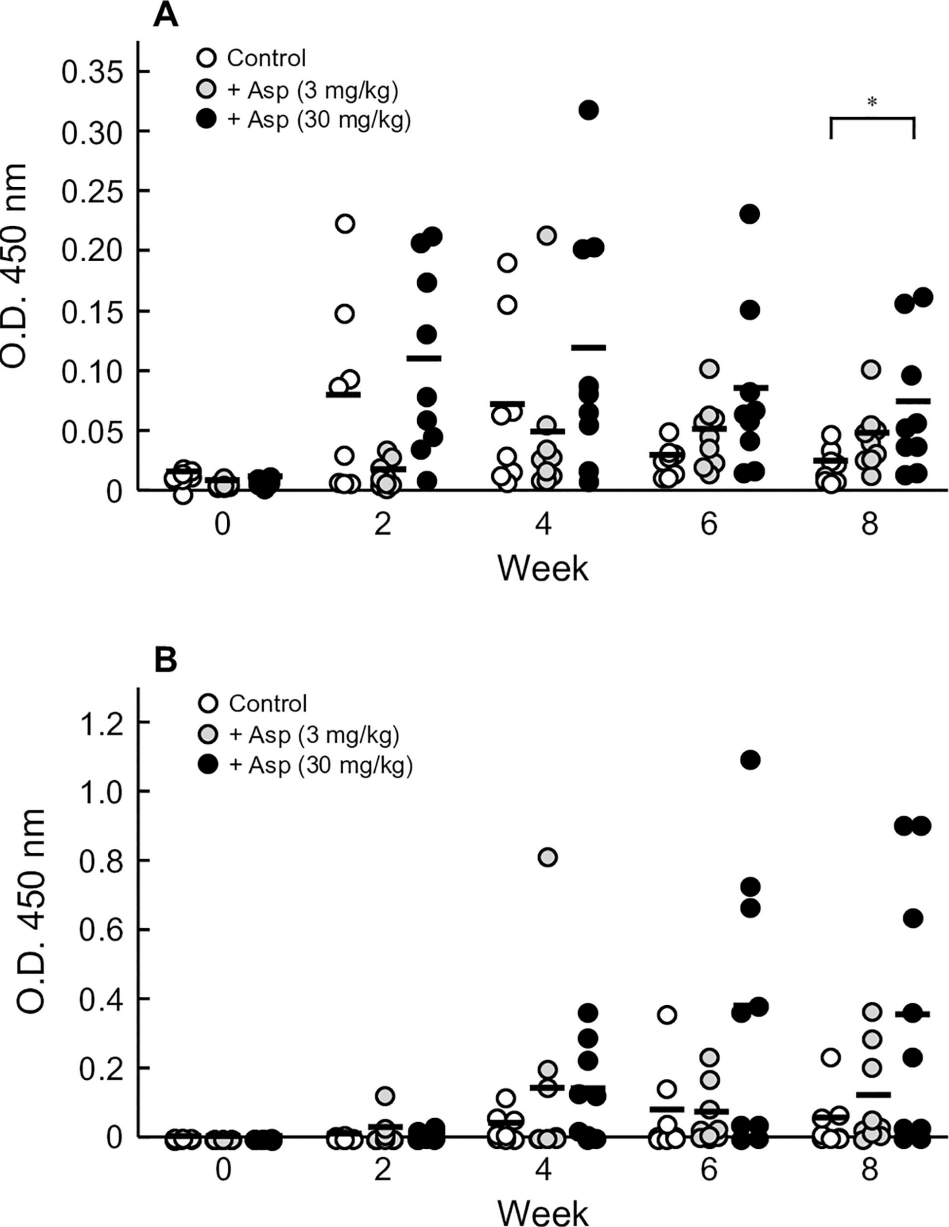
Effects of aspirin on oral sensitization to OVA in rats. Vehicle alone [phosphate-buffered saline (pH 7.4), Control] or vehicle containing aspirin (Asp, 3 or 30 mg/kg) was administered 30 min before oral administration of OVA at a dose of 50 mg/animal. These oral immunizations were repeated every other day for 8 weeks. Plasma levels of OVA-specific IgE (A) and IgG_1_ (B) Abs in rats were measured by ELISA. The optical densities measured at 450 nm in 10-fold- or 30,000-fold-diluted plasma are shown. Bars represent the mean values of eight to nine rats. *P < 0.05 compared with the Control group.

**Fig 3:**
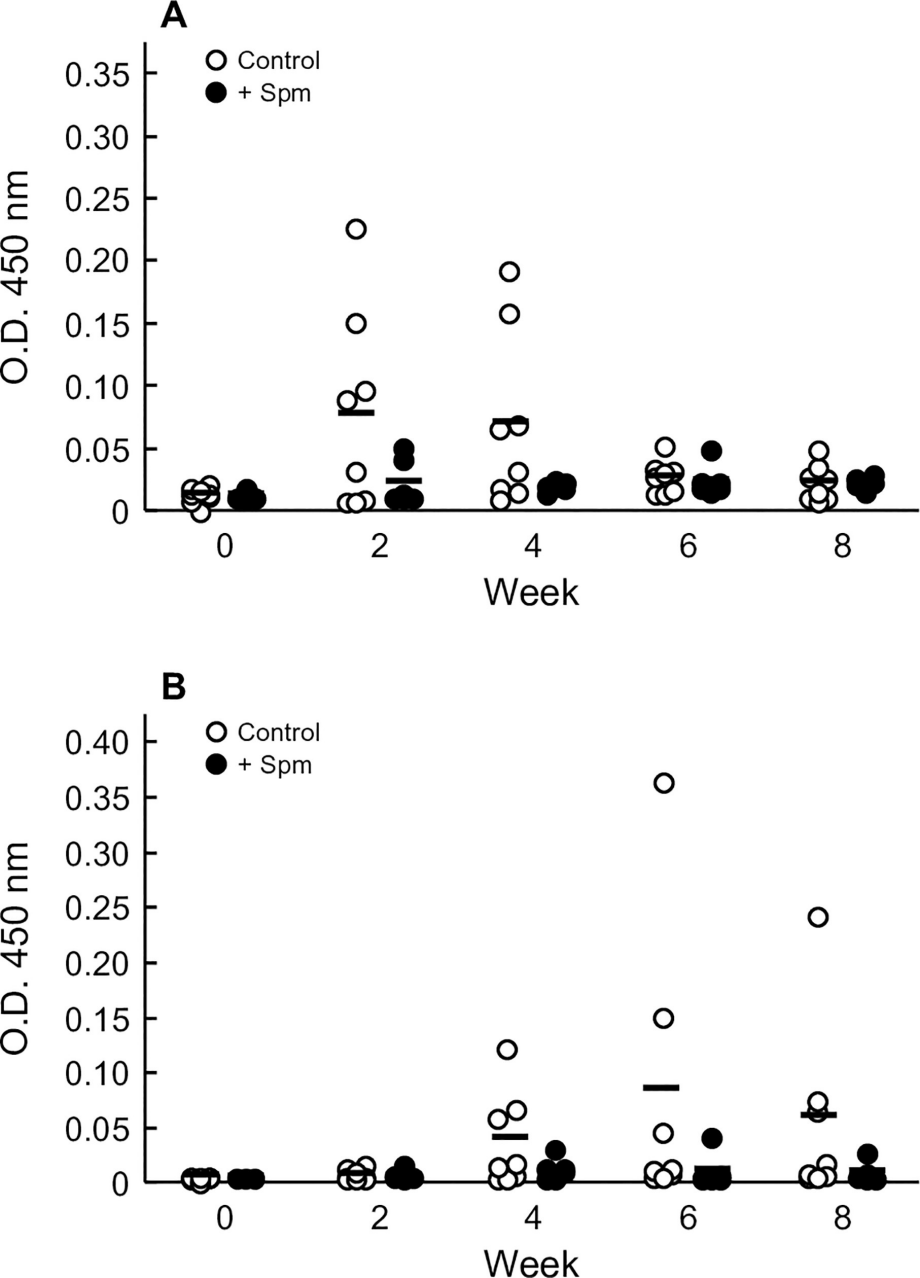
Effects of spermine on oral sensitization to OVA in rats. Vehicle alone [phosphate-buffered saline (pH 7.4), Control] was administered 30 min before oral administration of OVA at a dose of 50 mg/animal. Spermine (Spm, 5 mg) was orally administered with OVA (50 mg/kg) simultaneously. These oral immunizations were repeated every other day for 8 weeks. Plasma levels of OVA-specific IgE (A) and IgG_1_ (B) Abs in rats were measured by ELISA. The optical densities measured at 450 nm in 10-fold- or 30,000-fold-diluted plasma are shown. Bars represent the mean values of six to eight rats.

Plasma levels of OVA-specific IgE and IgG_1_ Abs were determined to evaluate the effects of aspirin and spermine on oral sensitization (Figs 2 and 3). When OVA was orally administered to rats every other day for 8 weeks, plasma levels of OVA-specific IgE Ab were increased at 2 weeks after initiation of sensitization (Figs 2A and 3A). Then, the elevated plasma levels of OVA-specific IgE Ab gradually decreased over time. The plasma levels of OVA-specific IgG_1_ Ab were also elevated in vehicle-treated rats, but the increase was slower than that of IgE Ab (Figs 2B and 3B). Low-dose aspirin exerted no significant effects on the plasma levels of OVA-specific IgE and IgG_1_ Abs (Fig 2). High-dose aspirin increased the plasma levels of OVA-specific IgE Ab after 8 weeks of treatment. Plasma levels of OVA-specific IgG_1_ Ab were also increased by high-dose aspirin at 6 and 8 weeks, although the difference was not statistically significant. In contrast, spermine did not affect the plasma level of either OVA-specific IgE or IgG_1_ Abs (Fig 3).

### Effects of NSAIDs on oral sensitization to OVA

**Fig 4:**
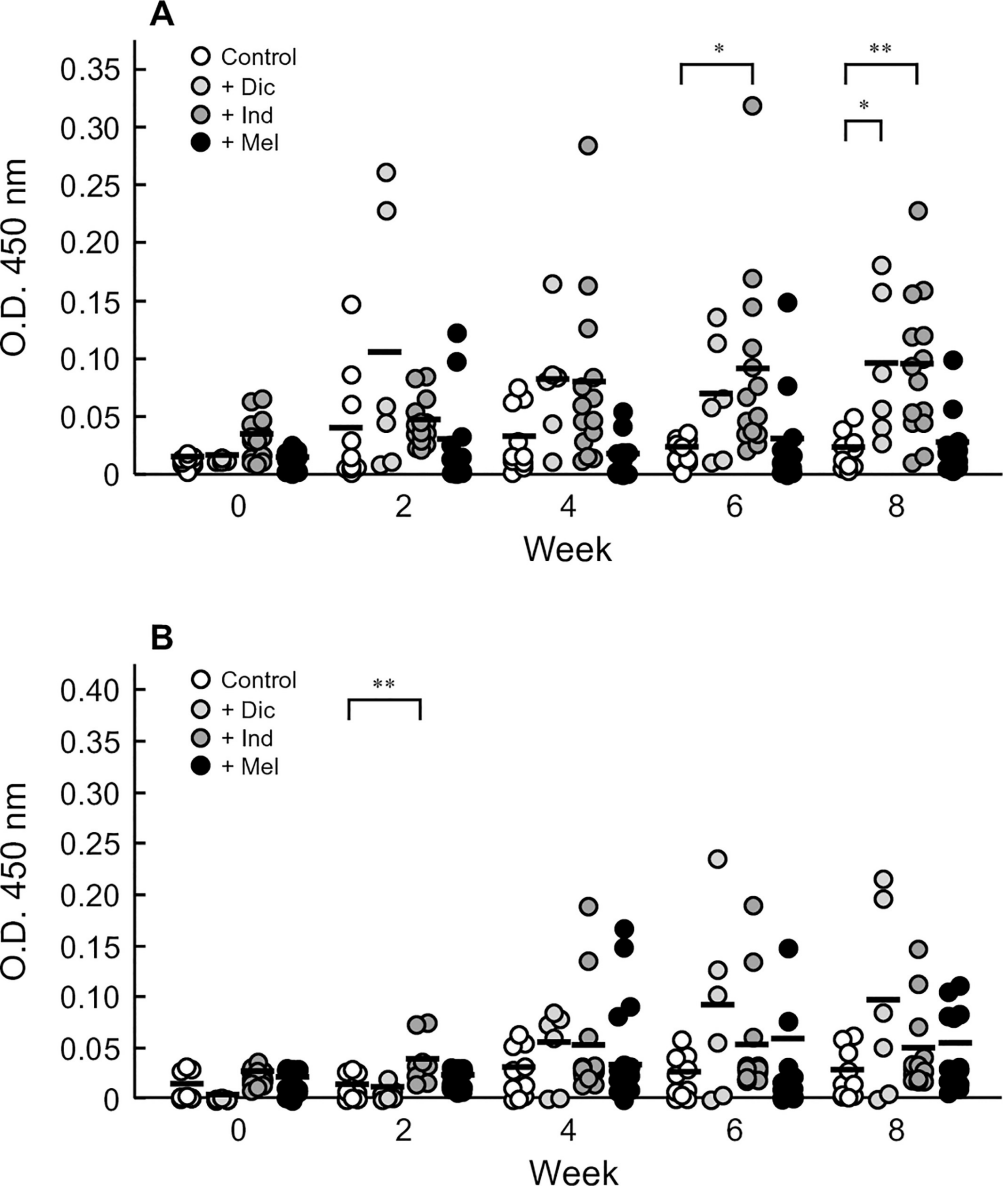
Effects of various NSAIDs on oral sensitization to OVA in rats. Vehicle alone [phosphate-buffered saline (pH 7.4), Control] or vehicle containing diclofenac (Dic, 1.5 mg/kg), indomethacin (Ind, 3 mg/kg), or meloxicam (Mel, 0.3 mg/kg) was administered 30 min before oral administration of OVA at a dose of 50 mg/animal. These oral immunizations were repeated every other day for 8 weeks. Levels of OVA-specific IgE (A) and IgG_1_ Abs (B) in the plasma of rats were measured by ELISA. The optical densities measured at 450 nm in 10-fold- or 30,000-fold-diluted plasma are shown. Bars represent the mean values of six to fourteen rats. *P < 0.05 and **P < 0.01 with respect to the Control group.

To evaluate the involvement of selective COX inhibition by NSAIDs in the oral sensitization to OVA, diclofenac, indomethacin, and meloxicam were tested. In diclofenac-treated rats, at 8 weeks, the plasma levels of OVA-specific IgE Ab were higher than those in control rats (Fig 4A), and the plasma levels of IgG_1_ Ab tended to be higher than those in control rats (Fig 4B). Indomethacin significantly increased the plasma levels of both OVA-specific IgE and IgG_1_ Abs, whereas meloxicam did not affect the Ab levels (Fig 4).

## Discussion

Previous reports have shown that aspirin induced and exacerbated allergic symptoms by promoting absorption of ingested allergens in rats and patients with IgE-mediated food allergies [7–9,11]. However, there are no reports regarding the effect of aspirin on the sensitization to ingested allergens. In this study, we showed that aspirin, diclofenac, and indomethacin enhanced the oral sensitization to OVA in rats.

In clinical settings, aspirin is used as an anti-inflammatory and anti-coagulant agent at doses of 0.5–1.5 g and 0.1–0.3 g, respectively. According to US Food and Drug Administration guidelines, these doses in humans correspond to 62–186 mg/kg and 12.4–37.2 mg/kg in rats, respectively [12]. In this study, aspirin was administered to rats at a dose of 30 mg/kg and 3 mg/kg. Similarly, diclofenac, indomethacin, and meloxicam are orally administered in clinical settings at doses of 25–100 mg, 25–75 mg and 10–15 mg, respectively. These doses in humans correspond to 3.1–12.4 mg/kg, 3.1–9.3 mg/kg and 1.24–1.86 mg/kg in rats, respectively [12]. In this study, diclofenac, indomethacin, and meloxicam were administered to rats at doses of 1.5 mg/kg, 3 mg/kg and 0.3 mg/kg, respectively. Thus, the doses of NSAIDs used in this study may have been smaller, but not significantly smaller, than those used clinically.

Aspirin enhanced the absorption of ingested OVA in a dose-dependent manner (Fig 1A). In this study, the plasma concentrations of OVA were determined by a sandwich ELISA, indicating that both intact and/or partially digested OVA might be detected in plasma. We previously demonstrated that OVA were absorbed from intestine as an intact form in the presence of protease inhibitors using in situ intestinal perfusion technique [5]. In addition, aspirin elicited and/or exacerbated the allergic symptoms by increasing oral absorptions of OVA [9]. These reports suggested that aspirin could enhance the oral absorption of OVA as an intact form at least partly. Furthermore, we previously reported that aspirin facilitated the permeability of macromolecules including OVA following impairment of the paracellular pathway [4,5]. Several reports have shown that aspirin induced intestinal barrier disruption due to the suppression of prostaglandin production by inhibiting COX-1 [13], oxidative stress [14], and/or modulation of tight junctional proteins [15,16]. Our previous report showed that diclofenac, a non-selective COX-1 and COX-2 inhibitor, facilitated the absorption of the egg-white allergen lysozyme, but meloxicam, a preferential COX-2 inhibitor, exerted no effects on its absorption [4]. In addition, coadministration of misoprostol (a synthetic prostaglandin-E1 analog) with aspirin ameliorated the aspirin-facilitated absorption of lysozyme to the same extent as that observed in untreated rats. Louis et al. [17] and Isobe et al. [18] reported that indomethacin (a preferential COX-1 inhibitor) increased OVA absorption. These results may suggest that aspirin facilitates the absorption of ingested allergens as a result of reduced prostaglandin production via inhibition of COX-1. Similar to aspirin, spermine increased the absorption of ingested OVA (Fig 1B). The mechanism of by which spermine facilitates OVA absorption is not completely understood. Sugita et al. reported that spermine increased the oral absorption of macromolecules such as dextran without causing severe epithelial damage [19]. They also suggested that interaction of the positively charged amino groups of spermine with the negative membrane components might influence intestinal permeability to macromolecules. Thus, the mechanisms for enhanced OVA absorption may be different between aspirin and spermine. However, Sugita et al. also showed that coadministration of OVA and spermine increased in plasma histamine levels, as well as OVA absorption, in mice sensitized with OVA compared with oral administration of OVA alone [20]. Thus, we consider that both spermine and aspirin could enhance the oral absorption of OVA as an intact form at least partly.

Aspirin enhanced the oral sensitization to and oral absorption of OVA in a dose-dependent manner (Fig 2). However, spermine did not affect oral sensitization to OVA, although it facilitated OVA absorption (Fig 3). These results suggest that enhanced oral sensitization to OVA cannot be ascribed only to increased absorption of OVA from the intestinal tract. The mechanisms underlying the aspirin-facilitated oral sensitization to OVA are not clear at present. However, two possible hypotheses may explain this finding. Dhuban et al. [21] reported that the proportion of interleukin (IL)-17-producing CD4+ T cells (Th 17) in children with food allergy is significantly lower than that in healthy subjects by flow cytometric analysis of CD4+ T cells. Moon et al. reported that aspirin inhibited the production of IL-17 from lung T cells as well as the in vitro Th17 cell polarization induced by IL-6 in a mouse model of asthma [22]. They also reported that aspirin could transform the adenosine receptors-mediated Th17-type cells into Th2-type inflammatory cells in the lung via the upregulation of adenosine and uric acid productions in the surrounding inflammatory cells [23]. Furthermore, there was one report that prostaglandin E2 can directly promote the differentiation and proinflammatory functions of human and murine Th17 cells [24]. Thus, aspirin might impair Th17 polarization, which controls the sensitization to allergens. In addition to IL-17, some cytokines, including thymic stromal lymphopoietin (TSLP), IL-33 and IL-25, are key factors for the development of allergic diseases, such as asthma and skin atopic dermatitis, that act by promoting Th2-type responses [25–30]. These cytokines are released from epithelial cells of the intestine and lung and keratinocytes activated by stimulation with allergens, cytokines, and protease [31,32]. Thus, aspirin might increase the oral sensitization to OVA by inducing these cytokines following impairment of intestinal epithelial cells. Furthermore, it has been reported that some leukotrienes (LTs) including cysteinyl LTs, LTB4 and LTC4 caused allergic sensitization via promotion of IL-33 in lung [33,34]. Thus, aspirin might also enhance the oral sensitization to OVA, resulting from increasing leukotriene production by COX inhibition. Further studies are necessary to elucidate the factors that affect the aspirin-facilitated sensitization to food allergens.

In conclusion, we demonstrated that a clinical dose of aspirin enhanced oral sensitization to OVA in a rat food-allergy model. Our study suggests that enhanced oral sensitization to OVA cannot be ascribed to increased absorption of OVA from the intestinal tract. Although the mechanisms underlying this enhancement of sensitization are still controversial, our study suggests that modification of cytokine production due to impairment of the intestinal barrier function and inhibition of cyclooxygenase-1 activity by aspirin may be involved. These findings shed new light on the pathophysiological mechanisms underlying the effects of medication on oral sensitization to food allergens.
